# A Systematic Review and Meta-Analysis of Strength Recovery Measured by Isokinetic Dynamometer Technology after Anterior Cruciate Ligament Reconstruction Using Quadriceps Tendon Autografts vs. Hamstring Tendon Autografts or Patellar Tendon Autografts

**DOI:** 10.3390/ijerph19116764

**Published:** 2022-06-01

**Authors:** Fahed Herbawi, Mario Lozano-Lozano, Maria Lopez-Garzon, Paula Postigo-Martin, Lucia Ortiz-Comino, Jose Luis Martin-Alguacil, Manuel Arroyo-Morales, Carolina Fernandez-Lao

**Affiliations:** 1 Department of Physical Therapy, Health Science Faculty, University of Granada, Avda de la Ilustración 60, 18016 Granada, Spain; fbherbawi@correo.ugr.es (F.H.); maloga@ugr.es (M.L.-G.); paulapostigo@ugr.es (P.P.-M.); luciaoc@ugr.es (L.O.-C.); marroyo@ugr.es (M.A.-M.); carolinafl@ugr.es (C.F.-L.); 2 Instituto de Investigación Biosanitaria ibs. GRANADA, 18016 Granada, Spain; 3 “Cuídate” Support Unit for Oncology Patients (UAPO-Cuíate), Sport and Health University Research Institute (iMUDS), 18010 Granada, Spain; 4 Department of Orthopaedic Surgery, Andalusian Mutuality of Soccer Players and Martín Gómez Clinic, 18005 Granada, Spain; joseluismartinalguacil@hotmail.com

**Keywords:** anterior cruciate ligament reconstruction, isokinetic test, quadriceps tendon autograft

## Abstract

Background: This systematic review and meta-analysis compared the isokinetic strength of the muscular knee joint between quadriceps tendon autografts (QTAs) and hamstring tendon autografts (HTAs) or patellar tendon autografts (PTAs) after anterior cruciate ligament (ACL) reconstruction by determining the isokinetic angular velocity and follow-up time points. The functional outcomes and knee stability at the same time points were also compared using isokinetic technology. Methods: Two independent reviewers searched the Medline (via PubMed search engine), Scopus, Web of Science and Cochrane Library databases to include full text comparative studies that assessed isokinetic strength test following ACL reconstruction. The DerSimonian and Laird method was used. Results: In total, ten studies were included; seven compared studies QTAs vs. HTAs, and three compared QTAs vs. PTAs. Five studies were included in the meta-analysis. Isokinetic strength data were reported 3, 6, 12 and 24 months after ACL reconstruction. Conclusions: The QTAs showed better and significant results with knee flexion compared with HTAs, similar results to PTAs at 6 and 12 months. While HTAs showed better and significant results with knee extension at 6 months and similar results at 12 months compared to QTAs. Furthermore, a standardized isokinetic strength test must be followed to achieve a more specific conclusion and better clinical comparison among participants.

## 1. Introduction

Anterior cruciate ligament (ACL) injury is a common knee injury with an incidence of between 32 and 80 cases per 100,000 inhabitants every year worldwide [1,2,3,4] and approximately 25,000 injuries per year in the United States [5]. Reconstruction of the ACL is the standard surgical method that aims to repair knee stability [6], improve both clinical and functional outcomes, achieve a rapid return to sport (RTS) [7,8,9] and reduce the potential risk of knee osteoarthritis [6,10]. Quadriceps tendon autografts (QTAs) have become more popular in the last 20 years because of their advantages over knee stability and muscle strength recovery [11,12,13,14,15]. Patellar tendon autografts (PTAs) and hamstring tendon autografts (HTAs) are the most commonly used autografts [6,16,17]. HTAs show good quadriceps recovery and less donor site morbidity but are associated with hamstring muscle deficits and slower rehabilitation processes [18,19]. PTAs offer the advantage of good hamstring recovery and a stable knee [20]. By contrast, PTAs have been associated with anterior knee pain and quadriceps strength deficiency [20,21]. Therefore, choosing ACL reconstruction autografts remains controversial because of their advantages and disadvantages.

Rehabilitation protocols play a significant role in RTS. Pre-surgery protocols comprise one phase and aim to achieve a 90% quadriceps limb symmetry index [22]. The post-surgery protocol comprises four to five phases over 6 months [22,23,24], forming the standard rehabilitation protocol [22,23,24]. However, evidence supports that the type of graft used in reconstruction alters the above phases. For example, reconstructed patients with HTAs delay their resisted hamstring exercise [19,22]. Therefore, studying the impact of each type of graft on rehabilitation protocols is warranted.

Rapid RTS is the desire for all athletes [25] and one of the most commonly used endpoints to evaluate the effectiveness of surgical techniques. In this sense, a systematic review and meta-analysis by Ardern et al. [26] reported that between 62% and 81% of injured athletes returned to their pre-injury level after surgery. Additionally, 44% returned to their competitive level [26]. Furthermore, RTS criteria are multifactorial and include time after surgery, muscle strength, and functional outcomes [9,22,27]. Functional outcomes are considered subjective methods to evaluate patient progress after rehabilitation protocols (e.g., the Lysholm, Tegner, International Knee Documentation Committee IDKC, and Cincinnati scores) [9,22,28]. Among all RTS criteria, muscle strength is the most important considered criterion [9,29], and the most commonly used methods for its evaluation are manual muscle testing [30], isometric strength tests [30] and isokinetic strength tests [31]. Furthermore, the isokinetic technology is considered “the gold standard” method for evaluating muscle strength, allowing the quantification of muscle strength through the determined angular velocity [31,32].

Zemach et al. [33] and Ypici et al. [34] reported that the use of different angular velocities led to different results [33,34]. Accordingly, Undheim et al. mentioned that no clear standardized isokinetic testing protocol was used in the published articles, limiting the quantitative comparison among published data [35]. Therefore, the homogeneity of the patient evaluation time points, isokinetic strength test protocol and instrument used is complex.

The current literature has revealed many studies comparing HTAs vs. PTAs. Kurz et al. [16] included 17 meta-analyses in their study comparing HTAs vs. PTAs regarding muscle strength, functional outcomes, and knee stability, among others [16]. Adam et al. [22] mentioned no differences in time to RTS between HTAs and PTAs. Additionally, previous systematic reviews compared QTAs vs. HTAs and PTAs and reported similar functional outcomes and better knee stability results with the QTA group. The current literature has revealed no evidence regarding RTS and strength recovery with QTAs vs. HTAs or PTAs. In this respect, a recent systematic review and meta-analysis by Johnston et al. [36] compared isokinetic and isometric tests between QTAs and PTAs or HTAs using the categorical angular velocity (low: 60°/s–90°/s and moderate: 160°/s–180°/s) and categorical follow-up periods (5–8, 9–15, 24, and 36–60 months). However, they did not compare a determined angular velocity or specific follow-up time points. Furthermore, no functional outcomes or knee stability were reported in the mentioned meta-analysis [36].

Therefore, given the substantial scientific evidence, as well as the difficulties posed by the meta-analyses mentioned above, we hypothesized that: (1) there would be a statistically significant differences between QTAs and HTAs or PTAs in the isokinetic strength test after ACL reconstruction; and (2) the current literature would show a difference between QTAs and HTAs or PTAs regarding knee stability and functional outcomes at the same follow-up points. Thus, this systematic review and meta-analysis aimed primarily to compare the isokinetic strength test of the quadriceps and hamstring muscles between QTAs and HTAs or PTAs after ACL reconstruction using the isokinetic angular velocity and follow-up time points. Additionally, as a secondary objective, we aimed to compare functional outcomes and knee stability at the same time points with isokinetic strength tests.

## 2. Materials and Methods

### 2.1. Protocol and Registration

This systematic review and meta-analysis was conducted in accordance with the Preferred Reporting Items for Systematic Reviews and Meta-Analyses (PRISMA) guidelines (Supplementary File S1) [37]. A detailed protocol for the systematic review was registered in the International Prospective Registry of Systematic Reviews (PROSPERO). It can be accessed with the code CRD42020191849. According to the PRISMA guidelines, the specific question posed for this review was, “which tendon autograft for anterior cruciate ligament reconstruction is better for strength recovery in athletes?”.

### 2.2. Study Eligibility

Studies were selected for inclusion based on the following criteria: (1) comparative studies; (2) participants aged between 16 and 45 years who had undergone ACL reconstruction surgery with a tendon autograft; (3) strength assessment using the isokinetic strength test; (4) accessible online full text (in any case, consideration was given to contacting the authors if access to the full text online was not available); and (5) studies published in English or Spanish. Studies such as reviews, case reports, monographs, guidelines, surveys, commentaries, conference papers and/or unpublished data were excluded, as well as studies performed on animals or in vitro.

### 2.3. Literature Search

The comprehensive search occurred between January and March 2021 in the Medline (via PubMed search engine), Scopus, Web of Science and Cochrane Library databases using the following search terms ((ACL reconstruction OR ACLR) AND (Quadriceps autograft OR quadriceps tendon OR QT) AND (isokinetic dynamometer OR isokinetic test)). To select information, the descriptors used were obtained from the Medical Subjects Heading (MeSH) database. The information was filtered using terms and keywords related to ACL reconstruction and rehabilitation procedures, combined with Boolean operators and search techniques adapted to each database. Additionally, the reference lists of retrieved reports were manually searched for additional references. The search equation was developed and replicated by two independent researchers (F.H. and C.F.-L.) autonomously and independently to ensure the reliability of the results.

### 2.4. Study Selection and Data Abstraction

This systematic review was developed independently by two authors (F.H. and C.F.-L.), who screened by title and abstract first and then by full text. Studies were evaluated in both phases according to the eligibility criteria mentioned above. If disagreement occurred between the reviewers, a third external reviewer (M.L.-L.) participated to decide whether to include or exclude the article. When completing both screenings, the search strategy was re-executed if additional studies were added to the literature and were retrieved for inclusion (latest search released on 1 March 2021).

The data abstraction process was performed by two researchers (F.H. and C.F.-L.). One first selected the data, and then the other verified this selection for accuracy. If any disagreement occurred, a third researcher (M.L.-L.) was asked to make a final decision. The collected data items were as follows: (1) first author; (2) year of publication; (3) study design; (4) clinical entity responsible for the study; (5) sample size; (6) type of intervention(s); (7) if applicable, details of control or comparison groups; and (8) main findings.

### 2.5. Risk of Bias Assessment

The risk of bias of the included studies in this systematic review was determined by two independent reviewers (F.H. and M.L.-L.) and was evaluated using specific scales depending on the type of study, following the instructions given by the Cochrane Handbook for Systematic Reviews of Intervention [38] and the National Institutes of Health (NIH) [39].

Randomized controlled trials (RCTs) were evaluated using the Revised Cochrane risk-of-bias tool for randomized trials (RoB 2) [40]. This common tool is used for randomized trials and has been updated in the last year. It assesses bias in five distinct domains (e.g., randomization process, intended interventions, missing data, measurements, and results). Observational studies were evaluated using the Cochrane’s tool Risk Of Bias In Non-Randomized Studies—of Interventions (ROBINS-I) with five level judgment criteria (low, moderate, serious, critical, and no information) for each domain. ROBINS-I tool assessed seven distinct domains (confounding, selection of participants, classification of interventions, deviations from intended, missing data, measurement of outcomes and selection of the reported results).

### 2.6. Data Analysis

To pool the results quantitatively and develop the proposed meta-analysis, as well as to generate corresponding forest plot graphs, STATA software was used (StataCorp. 2019; Stata Statistical Software: Release 16; StataCorp LLC, College Station, TX, USA). Only studies comparing the use of HTAs vs. QTAs or PTAs vs. QTAs and reporting valid isokinetic strength data obtained using an isokinetic dynamometer were included in this quantitative combination. Thus, a total of five studies were included in the meta-analysis [41,42,43,44,45]. The data were obtained from the tables or text of the articles, extracting the means and standard deviations (SD) of the follow-up values at 6 months, 12 months or 24 months. Where these data were not reported, the mean and SD were calculated from the available data based on the protocol previously published by Wan et al. [46]. The included studies were combined according to the follow-up, speed used (60°/s or 180°/s) and movement employed (knee flexion or knee extension). A random effects model of the DerSimonian and Laird method, which considers variations within and between studies, was used. Forest plots were developed to visualize individual study summaries and pooled estimates. Cochran’s Q statistic and the I^2^ value were used to study heterogeneity between studies. Cohen’s D was calculated for each of the original studies and an overall estimator, and a two-sided *p* value < 0.05 was considered statistically significant. Because of the low number of studies (<10), a more in-depth study of publication bias was not possible.

## 3. Results

### 3.1. General Overview

**Figure 1:**
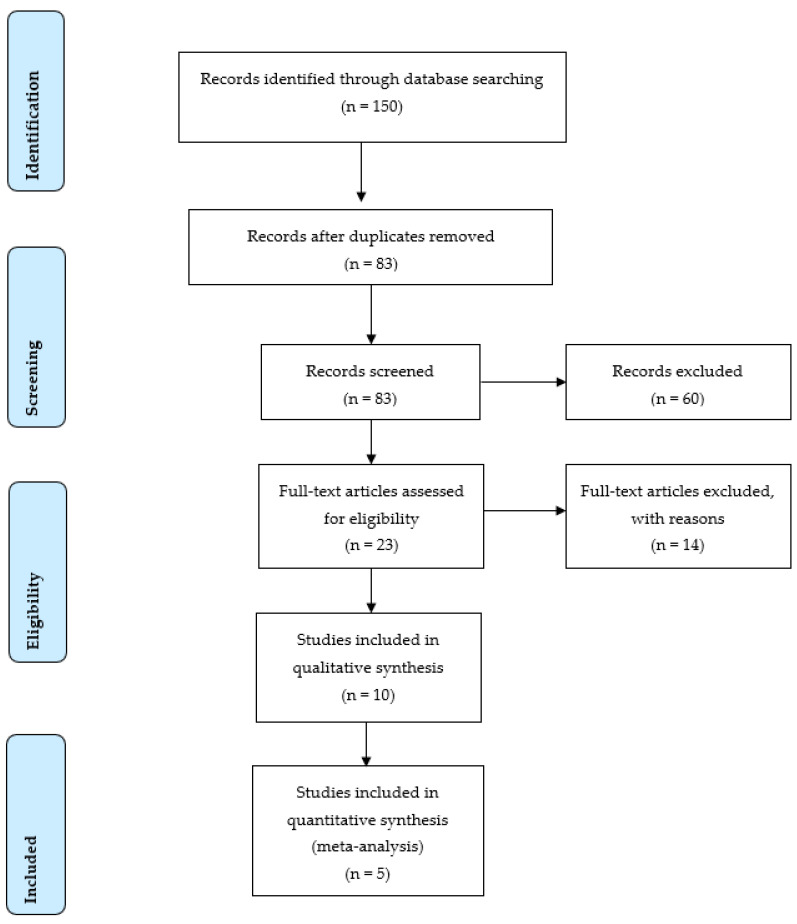
PRISMA Flow chart of search and study selection.

A total of 150 records were initially identified through database searching. Figure 1 shows the flow details of the selected trials in the different phases. The main reason for excluded articles was not comparing different autografts or not performing the isokinetic strength test. Finally, this systematic review included ten studies published between 2004 and 2020. Seven studies compared QTAs vs. HTAs [15,41,42,44,45,47,48], and three studies compared QTAs vs. PTAs [14,43,49]. Overall, the systematic review agglutinated 754 participants, of whom 376 had a QTA (271 male and 105 female; 27.01 ± 5.3 years), 267 had an HTA (187 male and 80 female; 22.46 ± 4.9 years), and 111 had a PTA (94 male and 17 female; 27.59 ± 7.5 years). Patient BMI was reported in seven studies including 440 patients, of whom 219 had a QTA with 23.68 ± 1.1 kg/m^2^, 206 had an HTA with 24.11 ± 0.41 kg/m^2^ and 15 had a PTA with 23.6 ± 0 kg/m^2^ [15,41,43,44,47,48,49].

Seven studies reported the meniscal tear percentage from the total sample (QTA = 50.03%, HTA = 43.27% and PTA = 60.54%) [14,15,41,42,43,45,47]. The time between diagnosis and surgery was reported in seven articles (QTA = 12.72 ± 7.01 months; HTA = 10.78 ± 6.29 months; PTA = 16.48 ± 10.96 months) [14,41,43,45,47,48,49]. The athletic status was only mentioned in four articles [14,41,44,45], three trials included only athletic patients [14,41,44], and Guney-Deniz et al. [45] did not include athletes. Regarding the study design, three were randomized control trials [14,44,47], one was a comparative study [43], five were cohort studies [15,41,44,47,48,49] and one was a cross-sectional study [45]. Table 1 displays the characteristics of the different intervention.

### 3.2. Risk of Bias

**Figure 2:**
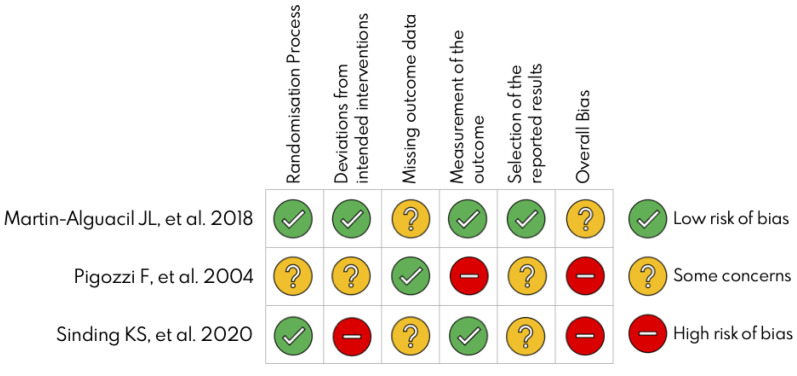
Cochrane collaboration risk of bias summary [14,44,47].

Two independent authors evaluated the risk of bias using the Cochrane risk-of-bias tool for randomized trials (RoB 2) for three randomized control trials [40] and Risk of Bias In Non-Randomized Studies of Interventions (ROBINS-I) for seven nonrandomized studies [50]. Two of three studies showed a high risk of bias, and the highest risk was in the “deviation from intended intervention” domain (Figure 2). However, most of the nonrandomized studies had a serious risk of bias, and the highest risk of bias was found in the “bias due to confounding” domain (Table 2).

### 3.3. Combined Outcomes

All the included investigations reported patient post-surgery outcomes with similar follow-up time points (one trial post 3 months [44], five studies post 6 months [14,42,43,44,48], two studies post 8 months [42,49], four studies post one year [15,43,44,47], and three studies post 2 years [15,43,49]).

#### 3.3.1. Three Months

Martin-Alguacil et al. [44] compared QTAs vs. HTAs 3 months after surgery and showed a significant increase in the quadriceps isokinetic strength (QIS) test in favor of the QTA group. No significant differences were reported between the groups in the hamstring isokinetic strength (HIS) test, functional outcome, or anteroposterior laxity.

#### 3.3.2. Six Months

**Figure 3:**
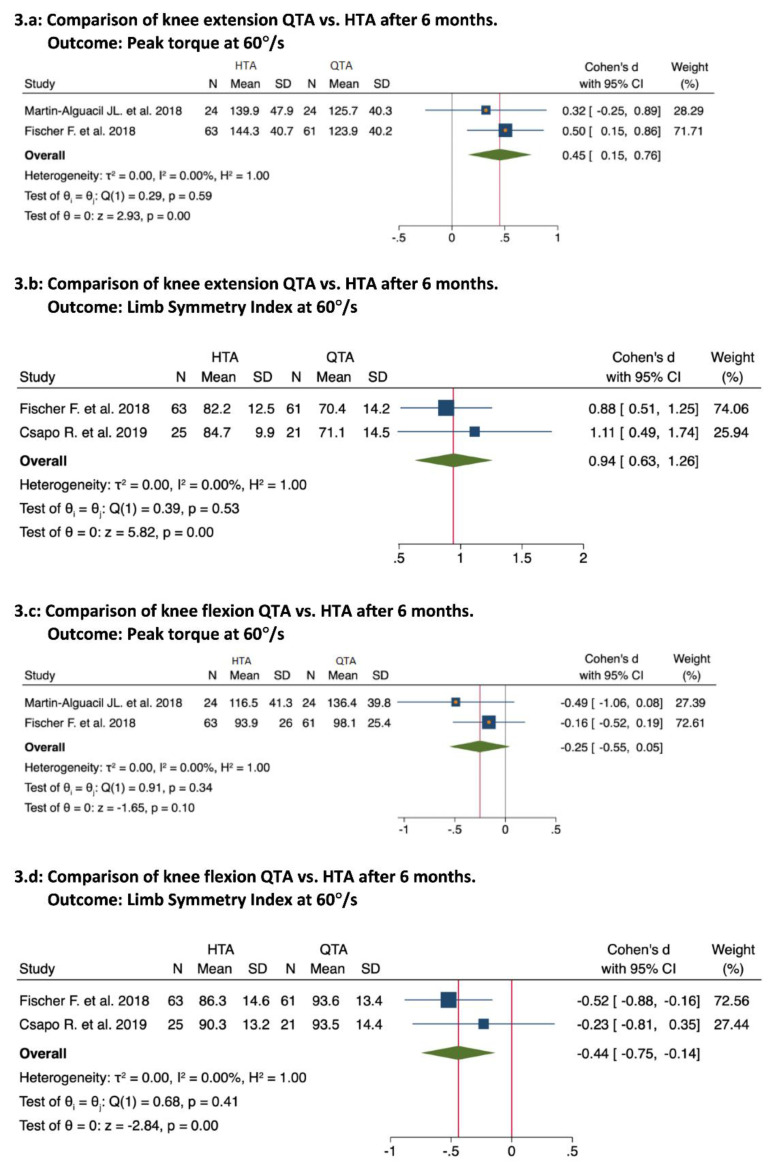
Forest plots for knee isokinetic strength test at 60°/s at 6 months [41,42,44].

Four studies compared QTAs vs. HTAs [41,42,44,48]; three mentioned better and significant results for the QTA group in the QIS test [41,42,44]. The pooled results were as follows: extension peak torque at 60°/s (0.45; 95% Confidence Interval (CI), 0.15 to 0.76; *p* = 0.00; I^2^ = 0%; Figure 3a; extension limb symmetry index (LSI) at 60°/s (0.94; 95% CI, 0.63 to 1.26; *p* = 0.00; I^2^ = 0%; Figure 3b) [41,42]. Similarly, the QTA group demonstrated better and significant results in the HIS test [48]. The pooled results were as follows: flexion peak torque at 60°/s (0.25; 95% CI, 0.05 to 0.55; *p* = 0.10; I^2^ = 0%; Figure 3c) [42,44]; flexion-LSI at 60°/s (0.44; 95% CI, −0.75 to −0.14; *p* = 0.00; I^2^ = 0%; Figure 3d) [41,42]. At the same evaluation point, two studies compared QTAs vs. PTAs [14,43]. Pigozzi et al. [14] reported better and significant results for QTA in the QIS and HIS tests. However, Han, H. et al. [43] demonstrated no significant differences between the groups in the QIS test or HIS test.

Regarding functional outcomes, two studies compared QTAs vs. HTAs. Martin-Alguacil et al. [44] showed no significant differences between the QTA and HTA groups. By contrast, Cavaignac et al. [48] showed significant differences between the QTA and HTA groups. No study has compared QTAs vs. PTAs regarding functional outcomes. Concerning knee stability, two studies compared QTAs vs. HTAs [44,48]. Cavaignac et al. reported better and significant results within the QTA group. However, Martin-Alguacil et al. [44] reported no differences between the groups. Additionally, only the study of Pigozzi et al. [14] reported knee stability between QTAs and HTAs and showed no significant differences between the autografts.

#### 3.3.3. Twelve Months

**Figure 4:**
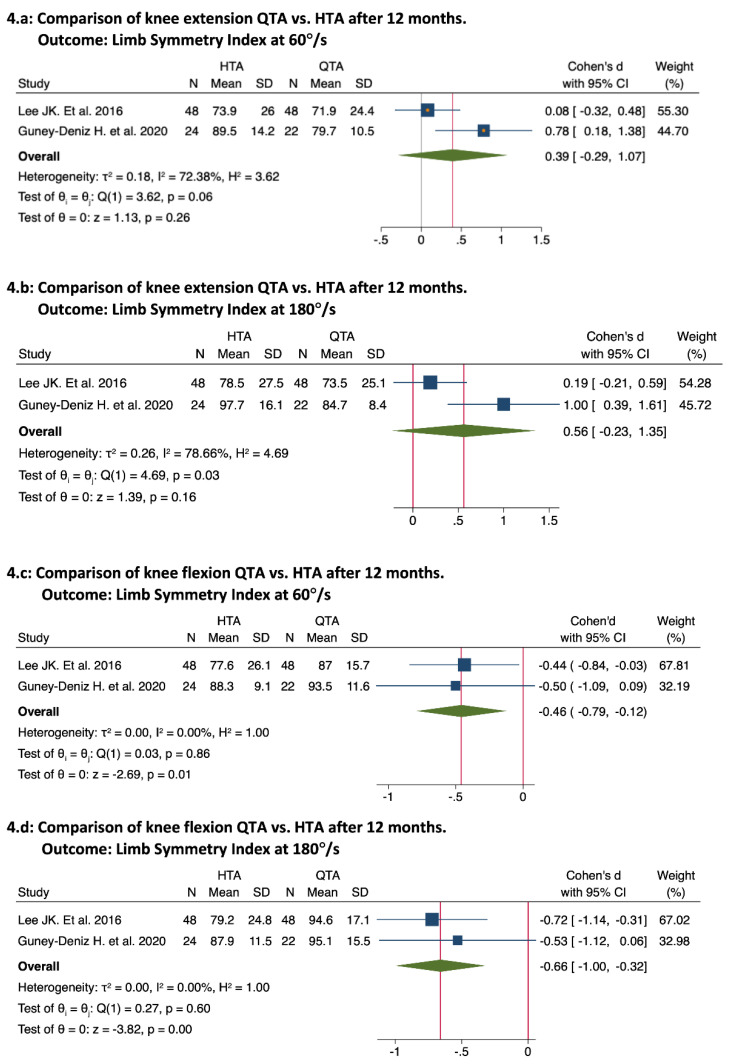
Forest plots for knee isokinetic strength test at 60°/s and 180°/s at 12 months [15,45].

The third evaluation point was approximately 12 months after surgery (12 months: [15,44,47,48]; 13.5 months: [45]). Four studies compared QTAs vs. HTAs [15,44,45,47], of which two reported no significant differences in the QIS test [15,44]. Two studies reported better and significant results with the HTA group in the QIS test [45,47]. The pooled results were as follows: knee extension LSI at 60°/s (39; 95% CI, −0.29 to 1.07; *p* = 0.26; I^2^ = 72.38%; Figure 4a) [15,45]; knee extension LSI at 180°/s (0.56; 95% CI, −0.23 to 1.35; *p* = 0.16; I^2^ = 78.66%; Figure 4b) [15,45]. Only one study showed significant results in the HIS test for the QTA group [15], and the HIS test showed no significant results in 3 studies [44,45,47]. The pooled results were as follows: knee flexion-LSI at 60°/s (0.46; 95% CI, −0.79 to −0.12; *p* = 0.01; I^2^ = 0%; Figure 4c) [15,45]; knee flexion-LSI at 180°/s (0.66; 95% CI, −1.00 to −0.32; *p* = 0.00; I^2^ = 0%; Figure 4d). Additionally, only Han et al. [43] compared QTAs vs. PTAs at 12 months and reported no significant differences between the groups. Finally, no significant differences were found between QTAs and HTAs regarding functional outcomes [15,44,45,47] or knee stability [15,44,47]. No study has compared QTAs vs. PTAs concerning functional outcomes or knee stability.

#### 3.3.4. Twenty-Four Months

The fourth evaluation point was approximately 24 months after surgery [15,43]. Only Lee et al. [15] compared QTAs vs. HTAs and reported no significant differences in the QIS test and significant differences in the HIS test for the QTA group. Similarly, Han et al. [43] compared QTA vs. PTA and mentioned no significant differences in the QIS test. Finally, no significant differences were found in functional outcomes or knee stability (1, 3) between QTAs and HTAs [15,44] or between QTAs and PTAs [43].

### 3.4. Return to Sport and Rehabilitation Protocols

Return to sport evaluation was different in the reviewed articles. Seven of ten studies described their rehabilitation protocol and return to sport criteria [14,15,42,43,44,45,47]. Post rehabilitation timing was mentioned in four studies [14,15,44]. Three studies had a 6-month accelerated rehabilitation program [14,15,44]. And one project had a 12-month nonaccelerated rehabilitation program [47]. However, 80% to 90% quadriceps strength recovery is considered a criterion to recover full activity and return to sport [14,43,47].

Post rehabilitation protocols were mentioned in seven studies with variations in their progression and phases [14,15,42,43,44,45,47]. Four rehabilitation programs started with pain and inflammation control in the first week [14,44,45,47], and full ROM was achieved between 3 and 6 weeks [14,15,42,43,44,45]. Although muscle strengthening started with static knee exercise in the first and second weeks [14,15,42,43,44,45], dynamic knee exercises were introduced between the second and fourth weeks [42,44,45,47]. Additionally, four protocols included closed kinetic chain exercises [14,42,44,45]. Martin-Alguacil et al. [44] and Lee et al. included open kinetic chain exercises. After that, four rehabilitation programs included running between 3 and 4 months. By contrast, three studies did not mention running in their protocols.

After the last phase of the rehabilitation protocols, RTS criteria were applied in six studies [14,15,43,44,45,47]. Time after surgery was mentioned in all criteria [14,15,43,44,45,47]. Guney-Deniz et al. [45] allowed RTS after 3 months. Five studies permitted RTS after 6 months [14,15,43,44]. By contrast, Sinding et al. [47] allowed RTS after one year. The second criterion was an isokinetic strength test of the injured limb of more than 80% to 90% of the non-injured limb [14,44,45]. The last criterion was a single-leg hop test of more than 80% to 90% of injured limbs [14,44,45]. Additionally, Hande et al. [45] was the only study to consider functional outcomes as RTS criteria.

## 4. Discussion

The main purpose of this systematic review and meta-analysis was to compare isokinetic strength tests, functional outcomes, and knee stability between QTAs and HTAs or PTAs after ACL reconstruction. Furthermore, this systematic review and meta-analysis added further quantitative analysis to previous systematic reviews [51,52] and included more studies than previously published studies [51,52]. Overall, 754 patients were evaluated from ten studies, and five of ten studies were included in the meta-analysis. The results suggest that ACL reconstructed patients with QTA showed better isokinetic strength results in the short term (e.g., 3 and 6 months). Additionally, they showed similar isokinetic strength results in the long term (e.g., 12 and 24 months) to HTAs and PTAs. Finally, our results showed similar results in functional outcomes and knee stability during short- and long-term evaluations between QTAs and HTAs or PTAs.

Comparing the isokinetic strength test between QTAs and HTAs or PTA, our results were similar to previous systematic reviews [36,51,52]. Additionally, our results were similar to a previous meta-analysis by Johnston et al. [36], where QTAs showed better isokinetic strength results during the short-term evaluation and similar results during the long-term evaluation. However, Johnston et al. [36] compared the isokinetic strength test using the categorical angular velocity (low: 60°/s–90°/s; moderate: 160°/s–180°/s) and categorical follow-up periods (5–8, 9–15, 24, and 36–60 months). We compared a determined angular velocity (60°/s or 180°/s) and determined follow-up time points (3, 6, 12 and 24 months). Furthermore, we could not compare the heterogeneity between the mentioned meta-analysis and our study because it was not reported. Additionally, they also compared only the peak torque of the LSI and did not compare that of the injured limb. In our study, we compared the peak torque from the injured limb and that of the LSI, revealing that the peak torque results for the uninjured limb contrast the peak torque results of the LSI [36]. Furthermore, the studies from Martin-Alguacil et al. [44] and Undheim et al. [35] have shown that the use of different angular velocity lead to statistical different results, which were not considered by the author of the previous meta-analysis [36]. Moreover, the mentioned meta-analysis has used downs and black scale to evaluate the risk of bias of the selected studies. This tool has been considered numerical quality assessment scale and, in our study, we have used RoB 2 and ROBINS-I from Cochrane Handbook for Systematic Reviews of Intervention. Five studies were excluded in the meta-analysis [14,43,47,48,49]. The main reasons were that Pigozzi et al. [14] did not report the isokinetic test angular velocity and Cavaignac et al. [48] reported the isokinetic angular velocity at 90°/s, preventing the formation of a meta-analysis group with other studies. Three studies were excluded because their follow-up time points did not form any meta-analysis group [43,47,49]. Finally, no meta-analysis subgroup comparing QTAs vs. PTAs was introduced because of the variations in the testing protocols or follow-up time points.

Regarding functional outcomes, similar to our results, a systematic review and meta-analysis by Hurly et al. [51] showed no significant differences between QTAs and HTAs or PTA. Additionally, Hurly et al. [51] reported functional outcomes with a mean of 24 months for HTA and 36 months for PTA, our results included the first 24 months post-surgery. However, this systematic review and meta-analysis did not match Ajrawat et al. [53] or Belk et al. [11], who reported better functional outcomes with QTAs vs. HTAs. This difference may be because both reviews included mostly nonrandomized or cohort studies. Finally, the functional outcome scores were similar among QTAs, HTAs and PTAs, but drawing a strong conclusion might be difficult because the data were reported using different functional outcomes (Lysholm, Tegner, IDKC, and Cincinnati scores) and different time points (6, 12, 26, and 36 months) [15,43,44,45,47,48].

Restoring knee stability is considered an important purpose of ACL reconstruction [54]. QTAs showed better knee stability than HTAs in a previous systematic review by Belk et al. [11]. However, the current systematic review and meta-analysis showed no difference in knee stability between QTAs and HTAs or PTAs. The systematic review by Belk et al. included eight studies (1 Level II, 7 Level III), none of which had a randomized controlled clinical trial RCT design. We showed similar results to the previous systematic review and meta-analysis by Mouarbes et al. [55] that analyzed 12 studies; of those, seven compared QTAs vs. HTAs, and five compared QTAs vs. HTAs. They reported no significant differences among the reconstructed autografts [55]. Additionally, knee stability might be affected by several factors in addition to the autograph type, such as the screw type, surgical procedure and aggressive postoperative rehabilitation [54]. Similar to the functional outcomes, a meta-analysis was not performed because of few matched studies [14,15,43,44,48].

This systematic review and meta-analysis showed some limitations. First, only five studies were included in the meta-analysis because of their methodological differences. Second, all the studies were included in the review despite their methodological characteristics. Third, the search, limited to English and Spanish languages, led to a potential publication bias. Fourth, some ACL reconstruction outcomes were not analyzed, such as the one-legged hope test or graft failure, because of the high variation among the studies. However, this study showed some strength points, reported according to the PRISMA guidelines. A risk of bias assessment was included, and a meta-analysis with low statistical heterogeneity was obtained because of the inclusion of determined subgroups.

We propose a standardized isokinetic strength test to ensure the comparison between further studies, as previous authors have recommended [35]. Such tests included five repetitions for knee flexion and another five for knee extension with one minute of rest between each test. Two angular velocities should be applied starting at 60°/s and then 180°/s. Additionally, patients should be seated with 85 degrees of hip flexion and 90 degrees of knee flexion. Furthermore, to ensure the comparison between testing protocols, standardized time points for evaluation (6, 12, and 24 months after surgery) may be useful. Indeed, all the tests may be applied to injured and uninjured limbs, allowing the examiner to report data on one limb and LSI.

## 5. Conclusions

This systematic review and meta-analysis adds further quantitative data analysis to previously published systematic reviews. The QTAs showed better and significant results in HIS compared with HTAs and similar results to PTAs at 3, 6 and 12 months. While HTAs showed a better and significant result in QIS at 6 months and similar results at 12 months compared to QTAs. This review showed similar results between QTAs and HTAs or PTA in functional outcomes and knee stability. Furthermore, a standardized isokinetic strength test must be followed to achieve a more specific conclusion and better clinical comparison among participants.

## Figures and Tables

**Table 1:** Characteristics of 10 studies included, according to the methodology used.

Author (Year) Study Design	Objective	Participants	Rehabilitation Procedures (Duration)	Outcomes Measures	Evaluation Follow-Up	Participants Gender *n* (%)	Principal Findings
Cavaignac, E., et al., 2017 [48] Cohort study	To compare isokinetic strength test of HTA and QTA, stability, functional outcomes scores, anterior knee pain and reoperation rate.	95 patients QTA; *n* = 45; 32.1 ± 8 years HTA; *n* = 41; 30.9 ± 9 years.	–	Functional outcome (KOOS, Tegner and IKDC), Joint stability KT-1000, Lachman, pivot shift), anterior knee pain (Shelbourne-Trumper score), and isokinetic strength.	6 and 43 months post-surgery	QTA: 55% male HTA: 58% male	The use of a QTA graft in ACL reconstruction leads to equal or better functional outcomes than does the use of an HTA graft, without affecting morbidity.
Csapo, R., et al., 2019 [41] Retrospective cohort study	To assess the fitness of elite alpine skiers during recovery from ACL reconstruction and changes in performance level after return to competition.	46 athletes; QTA; *n* = 21; HTA; *n* = 25.	–	Isokinetic dynamometry, back in action test battery (knee function after ACL recovery), VAS Functional outcome (Lysholm score, Tegner activity scale)	15 days, 6, 12, and 24 months post-surgery	20 male vs. 26 female	The rate of recovery of knee extensor muscle function may be slower following ACL reconstruction using QTA. On overage, athletes returned to competition within one year after surgery and succeeded in surpassing their baseline performance level within the first year after return to competition.
Fischer, F., et al., 2018 [42] Randomized Controlled Study	To compare isokinetic strength test for Quadriceps in who received either QTA or HTA autografts at two-time intervals within the first year after surgery.	124 patients QTA; *n* = 61; 21.7 ± 7.4 years, HTA; *n* =63; 21.5 ± 6.9 years.	Isometric and closed chain exercises, bicycling running and sport-specific exercises post-operatively.	Isokinetic strength test.	5.5- and 7.6-months post-surgery	QTA: male 34 (55.7). HTA: male 47 (74.6)	ACL reconstruction with a QTA autograft have a significantly higher H/Q ratio within one year after surgery compared to the HTA group.
Guney-Deniz, H., et al., 2020 [45] Cross-sectional, case–control study	To compare isokinetic strength test, the active joint position sense and knee functions in individuals who had anterior cruciate ligament (ACL) reconstruction with QTA, HTA, TAA and healthy individuals.	67 subjects QTA; *n* = 22; 27.8 ± 2.8 years, HTA; *n* = 24; 26.7 ± 4.6 years, ATT; *n* = 21 26.4 ± 5.5 years, Control; *n* = 20 28.7 ± 3.1 years.	Post-operative protocol includes progressive quadriceps femoris strengthening with neuromuscular electrical stimulation, and neuromuscular control exercise training.	Isokinetic strength test and active joint position sense assessments	13.5 months post-surgery	–	Knee proprioception deficits and impaired muscle strength were evident among patients at a mean 13.5 months post-ACL reconstruction compared with healthy controls. QTA group may be more likely to actively over-estimate knee position near terminal extension.
Han, H.S., et al., 2008 [43] Retrospective comparative study	To compare the clinical outcomes of anterior cruciate ligament reconstructions using QTA and PTA autografts.	144 patients QTA; *n* = 72; 27.8 (15–51) * years, PTA; *n* = 72; 27.8 (15–51) * years	–	knee stability (KT-1000), Functional outcome (Lysholm and IKDC) and Isokinetic strength test.	Pre-surgery, 6, 12 and 24 months	QTA: 68 male vs PTA: 68 male.	QTA group showed clinical outcomes comparable to PTA group with anterior knee pain beingless severe in the former. The data indicate the quadriceps tendon can be a good alternative graft choice.
Hunnicut, J.K., et al., 2019 [49] Cohort Study	To compare quadriceps recovery and functional outcomes in patients with QTA versus PTA autografts.	30 patients QTA; *n* = 15; 25.0 (14.0–41.0) years PTA; *n* = 15; 18.0 (15.0–32.0) years	–	Isometric and isokinetic strength testcentral Activation, MRI, Spatiotemporal Gait Hop Test and Functional outcome (IKDC, Lysholm, and KOOS)	8 months post-surgery	QTA: male 12 vs. PTA: male 7	Patients with QTA autografts demonstrated similar short-term quadriceps recovery and postsurgical outcomes compared with patients with PTA autografts.
Lee, J.K., et al., 2016 [15] Cohort study	To compare functional outcomes and knee joint stability of anatomic ACL reconstruction with double-bundle hamstring tendon and bone–quadriceps tendon autografts	96 patients QTA; *n* = 48; 31.1 (17–57) * years, HTA; *n* = 48; 29.9 (17–58) * years	Post-operative protocol includes quadriceps-strengthening, continuous passive motion, open kinetic chain exercise and kinetic exercises. (6 months)	Knee stability (Manual laxity test, KT-2000) Functional outcome (IKDC, Tegner activity score, modified Lysholm score), anterior knee pain questionnaire, Isokinetic strength test and tunnel position evaluation by quadrant method.	Pre-surgery and 6 weeks, 3, 12 and 24 months post-surgery	QTA: male 44. PTA: male 44	QTA group showed similar knee stability and functional outcomes when compared with the HTA autograft. Better flexor muscle strength recovery was found in the QTA, indicating a potential advantage of the QTA autograft in ACL reconstruction.
Martín-Alguacil, J.L., et al., 2018 [44] Randomized Controlled Study	To compare the strength recovery and functional outcomes of an anatomic single bundle reconstruction with QTA and HTA autografts in competitive soccer players.	51 participants QTA; *n* = 26; 18.7 ± 3.6 years, HTA; *n* = 25; 19.2 ± 3.6 years.	Both groups followed the same pre-and-post rehabilitation protocol based on muscular strength, endurance and neuromuscular control. (24 weeks)	Isokinetic strength test Function outcome (Lysholm knee score and Cincinnati Knee Rating System) and knee stability with KT-2000.	Pre-surgery and 3, 6, 12 and 24 months post-surgery	QTA: male 23 (88.5). HTA: male 16 (54.0)	QTA group showed similar functional outcome results with a better isokinetic H/Q ratio compared to HTA group at 12 months of follow-up in soccer players.
Pigozzi E., et al., 2004 [14] Prospective randomized study	To compare the isokinetic recovery of thigh strength after ACL reconstruction by using patellar or quadriceps tendon as a graft.	48 patients QTA; *n* = 24; 33 (21–47) years PTA; *n* = 24 35 (23–41) * years	Post rehabilitation program: continuous passive motion, walking, swimming, cycling and running at the end of 6 months. (6 months)	Counter movement jump, leg press, knee stability (KT-1000) and isokinetic strength tests.	Pre-surgery and 6 months post-surgery.	QTA: 17 male vs. PTA: 19 male	Significant improvement of the lower limb strength deficit using QTA compared to PTA that could encourage the use of QTA in order to achieve an easier rehabilitation and a faster Return to sport.
Sinding, K.S., et al., 2020 [47] Prospective randomized controlled clinical trial	To investigate the effects of QTA vs. HTA on thigh muscle strength and functional capacity, and a patient-reported outcome 1 year after ACL-R, and to compare the results to healthy controls.	150 patients QTA; *n* = 50; 128.7 ± 6.4 years, HTA; *n* = 50; 28.3 ± 6.2 years, CON; *n* = 50; 28.3 ± 6.2 years	Post rehabilitation program: days 1–14: full support to pain threshold, free movement, no bandages; weeks 3–12: frequent movement exercises supervised by a physiotherapist, bicycle ergometer, full weight bearing; months 4–9: running allowed; months 10–12: contact sports. allowed. (12 months)	Isokinetic strength test, one leg hop test and Functional outcome with IKDC	12.5 months post-surgery	QTA: male 25 (60%) HTA: male 23 (53%) vs. CON: male 27 (54%)	HTA leading to impairments of knee extensor and knee flexor muscle strength, while QTA results in more pronounced impairments of knee extensor only. Functional capacity and functional outcome was unaffected by autograft type, with the former showing impairment compared to healthy controls.

ATT, tibialis anterior tendon; HTA hamstring tendon autograft; H/Q, Hamstring/quadriceps; IKDC, International Knee Documentation Committee; KOOS, Osteoarthritis Outcome Score; MRI, Cross-sectional Area; PTA, patellar tendon autograft; QTA, quadriceps tendon autograft; VAS, visual analogy scale. * Median (range). –: None.

**Table 2:** Robins-I scale for the risk of bias assessment of non-randomized studies.

	D1	D2	D3	D4	D5	D6	D7	Overall Judgement
Cavaignac E., et al., 2017 [48]	Serious	Low	Low	Low	Serious	Low	Low	Serious
Csapo R., et al., 2019 [41]	Moderate	Moderate	Low	Low	Low	Low	Serious	Serious
Fischer F., et al., 2018 [42]	Moderate	Low	Low	Low	Low	Low	Low	Moderate
Han H.S., et al., 2008 [43]	Serious	Low	Low	Low	Critical	Low	Moderate	Critical
Guney-Deniz H., et al., 2020 [45]	Serious	Low	Low	Low	Low	Low	Low	Serious
Hunnicutt J.L., et al., 2019 [49]	Serious	Low	Low	Low	Low	Low	Moderate	Serious
Lee J.K., et al., 2016 [15]	Serious	Moderate	Low	Low	Low	Low	Moderate	Serious

D1, Bias due to confounding; D2, Bias in the selection of participants into the study; D3, Bias in the classification of interventions; D4, Bias due to deviations from intended; D5, Bias due to missing data; D6, Bias in the measurement of outcomes; D7, Bias in selection of the reported results. Background color caption: green = low risk of bias; yellow = moderate risk of bias; orange = serious risk of bias; and red = critical risk of bias.

## Data Availability

Raw data from data analysis are available upon reasonable request by contacting the corresponding author.

## Supplementary Materials

The following supporting information can be downloaded at: https://www.mdpi.com/article/10.3390/ijerph19116764/s1, Supplementary File S1 are supplementary data to this article.
